# Comparison of Tuberculin Skin Testing and Interferon-γ Release Assays in Predicting Tuberculosis Disease

**DOI:** 10.1001/jamanetworkopen.2024.4769

**Published:** 2024-04-03

**Authors:** Tracy Ayers, Andrew N. Hill, Julia Raykin, Sarita Mohanty, Robert W. Belknap, Richard Brostrom, Renuka Khurana, Michael Lauzardo, Thaddeus L. Miller, Masahiro Narita, April C. Pettit, Alexandra Pyan, Katya L. Salcedo, Araxi Polony, Jennifer Flood

**Affiliations:** 1 Division of Tuberculosis Elimination, National Center for HIV, Viral Hepatitis, STD, and TB Prevention, Centers for Disease Control and Prevention, Atlanta, Georgia; 2 Peraton, Reston, Virginia; 3 Public Health Institute at Denver Health, Denver, Colorado; 4 Tuberculosis Control Program, Hawai’i Department of Health, Honolulu; 5 Maricopa County Department of Public Health, Phoenix, Arizona; 6 Department of Medicine, University of Florida College of Medicine, Gainesville; 7 Department of Health Behavior and Health Systems, University of North Texas Health Science Center, Fort Worth; 8 Public Health—Seattle and King County, Seattle, Washington; 9 Division of Pulmonary, Critical Care, and Sleep Medicine, University of Washington, Seattle; 10 Division of Infectious Diseases, Vanderbilt University Medical Center, Nashville, Tennessee; 11 Maryland Department of Health, Baltimore; 12 Tuberculosis Control Branch, Division of Communicable Disease Control, Center for Infectious Diseases, California Department of Public Health, Richmond

## Abstract

**Importance:**

Elimination of tuberculosis (TB) disease in the US hinges on the ability of tests to detect individual risk of developing disease to inform prevention. The relative performance of 3 available TB tests—the tuberculin skin test (TST) and 2 interferon-γ release assays (IGRAs; QuantiFERON-TB Gold In-Tube [QFT-GIT] and SPOT.TB [TSPOT])—in predicting TB disease development in the US remains unknown.

**Objective:**

To compare the performance of the TST with the QFT-GIT and TSPOT IGRAs in predicting TB disease in high-risk populations.

**Design, Setting, and Participants:**

This prospective diagnostic study included participants at high risk of TB infection (TBI) or progression to TB disease at 10 US sites between 2012 and 2020. Participants of any age who had close contact with a case patient with infectious TB, were born in a country with medium or high TB incidence, had traveled recently to a high-incidence country, were living with HIV infection, or were from a population with a high local prevalence were enrolled from July 12, 2012, through May 5, 2017. Participants were assessed for 2 years after enrollment and through registry matches until the study end date (November 15, 2020). Data analysis was performed in June 2023.

**Exposures:**

At enrollment, participants were concurrently tested with 2 IGRAs (QFT-GIT from Qiagen and TSPOT from Oxford Immunotec) and the TST. Participants were classified as case patients with incident TB disease when diagnosed more than 30 days from enrollment.

**Main Outcomes and Measures:**

Estimated positive predictive value (PPV) ratios from generalized estimating equation models were used to compare test performance in predicting incident TB. Incremental changes in PPV were estimated to determine whether predictive performance significantly improved with the addition of a second test. Case patients with prevalent TB were examined in sensitivity analysis.

**Results:**

A total of 22 020 eligible participants were included in this study. Their median age was 32 (range, 0-102) years, more than half (51.2%) were male, and the median follow-up was 6.4 (range, 0.2-8.3) years. Most participants (82.0%) were born outside the US, and 9.6% were close contacts. Tuberculosis disease was identified in 129 case patients (0.6%): 42 (0.2%) had incident TB and 87 (0.4%) had prevalent TB. The TSPOT and QFT-GIT assays performed significantly better than the TST (PPV ratio, 1.65 [95% CI, 1.35-2.02] and 1.47 [95% CI, 1.22-1.77], respectively). The incremental gain in PPV, given a positive TST result, was statistically significant for positive QFT-GIT and TSPOT results (1.64 [95% CI, 1.40-1.93] and 1.94 [95% CI, 1.65-2.27], respectively).

**Conclusions and Relevance:**

In this diagnostic study assessing predictive value, IGRAs demonstrated superior performance for predicting incident TB compared with the TST. Interferon-γ release assays provided a statistically significant incremental improvement in PPV when a positive TST result was known. These findings suggest that IGRA performance may enhance decisions to treat TBI and prevent TB.

## Key Points

**Question:**

What is the relative performance of 3 clinically available tuberculosis (TB) tests in predicting TB disease development in the low-incidence setting of the US?

**Findings:**

In this diagnostic study of 22 020 participants, 2 US-approved interferon-γ release assays (IGRAs) demonstrated significantly superior performance in predicting progression to TB disease compared with the tuberculin skin test.

**Meaning:**

These findings suggest that IGRAs should be used to guide clinical decisions on prevention of TB disease.

## Teaser

This diagnostic study compares the performance of tuberculin skin testing and interferon-γ release assays in predicting development of tuberculosis (TB) disease in the US.

## Introduction

In the US, most tuberculosis (TB) cases arise from the large group of approximately 13 million asymptomatic individuals with TB infection (TBI), also known as latent TBI, which can progress to active TB disease.^1,2^ Although numerous studies have demonstrated the efficacy of TBI treatment to prevent progression to TB disease,^3,4,5^ optimal TBI tests to identify individuals at risk of TB disease are needed.^6^ The US Food and Drug Administration (FDA) has approved 2 test types: the tuberculin skin test (TST) and IFN-γ release assays (IGRAs), including QuantiFERON-TB Gold In-Tube (QFT-GIT; Qiagen) and T-SPOT.TB (TSPOT; Oxford Immunotec). All 3 are indirect tests that measure the immune response to TBI.^7^

Few studies have compared the performance of all 3 tests head to head in predicting progression to TB disease. At initiation of the present study in 2012, most published studies comparing TBI tests were limited to describing concordance. More recent studies have examined the ability of tests to predict future TB disease. However, these studies were limited in size, did not examine all 3 tests, or did not reflect low-incidence US settings.^8,9,10,11,12,13,14,15^ Limited evidence describes test use in clinical decision-making.^16,17,18^ In 2017, the American Thoracic Society (ATS), the Infectious Diseases Society of America (IDSA), and the Centers for Disease Control and Prevention (CDC) released graded recommendations for TB tests, with IGRAs being the preferred test for most people.^19^ Despite the preference for IGRAs within the US, situations in which a TST result is known at the point of care are common.^20^ Therefore, it is important to understand whether there is added value in performing additional testing.

The present study’s primary objective was to determine the ability of each test to predict progression to TB disease in TB clinics in the US. This study sought to improve on previous studies by including all 3 commercially available tests (TST, TSPOT, and QFT-GIT) in a head-to-head comparison within a large cohort of US individuals at high risk of TB disease. A secondary objective was to evaluate whether there was an increase in positive predictive value (PPV) with further testing, given an initial TST or IGRA result.

## Methods

### Study Design and Population

The CDC conducted this prospective diagnostic study in partnership with 10 public health TB programs. The study was approved by the CDC and local institutional review boards (45 CFR part 46 and 21 CFR part 56) and was registered at ClinicalTrials.gov (identifier NCT01622140). All participants provided written informed consent or assent, and those aged younger than 18 years had written parental or guardian permission. The study followed the Standards for Reporting Diagnostic Accuracy Studies (STARD) reporting guideline.

Participants were enrolled from 18 clinics starting July 12, 2012, through May 5, 2017, and were followed until the study end date (November 15, 2020). Participants were eligible for enrollment if they were a close contact (≥8 hours in a week) of an individual with smear-positive TB disease, were born in a country with medium or high TB incidence,^21^ were a recent immigrant (≤5 years) from a country with medium or high TB incidence,^21^ were a visitor (≥30 days) in the previous 5 years to countries with a medium or high TB incidence,^21^ were living with HIV infection, or were a member of a local population with documented TBI prevalence of 25% or greater. Participants were excluded if they had known anaphylaxis to tuberculin, were currently receiving treatment for TBI or TB disease, were scheduled to permanently leave the US within 2 years of enrollment, or were foster children.

Participants were tested with the TST, QFT-GIT, and TSPOT at study entry. None of these tests were considered the reference standard, and all were evaluated for their ability to predict progression to TB disease. Blood samples for the QFT-GIT and TSPOT were processed and reported according to the manufacturers’ instructions using the standard US cutoffs. Additional study procedure details have been published previously.^22,23^ The TSPOT borderline values (spot counts of 5, 6, or 7) were treated as negative results. The TSTs were read 44 to 76 hours after placement and interpreted as positive according to CDC criteria for classifying TST reactions.^24^ For all 3 tests, if a result was reported as invalid or indeterminate, it was treated as missing data for that individual test.

During the enrollment interview, participants self-reported vaccination status, medical histories, and demographic and epidemiologic risk factors. Race and ethnicity was evaluated as a known risk characteristic of TB disease. Participants self-reported race and ethnicity as American Indian or Alaska Native, Asian, Black or African American, Hispanic or Latino, Native Hawaiian or Other Pacific Islander, White, other race or ethnicity (not defined and often selected for multiple races or ethnicities), or did not know or declined to answer. Descriptive analysis was performed on all demographic variables. Participants with at least 1 positive test result were actively followed by telephone or with in-person visits every 6 months for the first 2 years after enrollment. All participants were followed via biannual registry matching through November 15, 2020, to assess for TB disease development.

### Study Definitions

#### Confirmed TB Disease

Possible case patients with TB disease were identified when a participant developed clinical signs or symptoms or when test results indicated possible TB. Possible case patients with TB were then verified by medical review and were considered to have confirmed TB disease if they met criteria for the US Report of Verified Case of Tuberculosis.^25^ Confirmed incident TB case outcome was considered the reference standard for comparing the performance of the 3 tests.

#### Prevalent TB

Individuals identified with confirmed TB disease at enrollment were not eligible for this study. However, some participants were identified with TB disease soon after enrollment (ie, ≤30 days) and were classified as case patients with prevalent TB. These case patients were excluded from the primary analyses and included in sensitivity analyses.

#### Study Outcomes: Incident TB and TB Disease Not Identified

Participants were classified as case patients with incident TB disease when diagnosed more than 30 days after enrollment. Participants who met the study enrollment criteria but were not identified as having prevalent or incident TB disease continued to be followed until the study end date (November 15, 2020). If TB disease remained undetected at study close, these participants’ outcome was labeled as TB disease not identified.

#### TBI Treatment Status

Decisions to initiate TBI treatment were determined by local practice. This study captured whether any TBI treatment was started and whether treatment was considered completed as determined by the clinician. Any participant with at least 1 positive test result who never started or completed TBI treatment was considered untreated.

### Statistical Analysis

The full analytic population was defined as participants who consented to study participation, met the eligibility criteria, and completed the enrollment interview. We compared demographic, epidemiologic, and self-reported medical histories between case patients with TB and those without TB identified using χ^2^ or Fisher exact tests. For case patients with incident TB, we calculated time to TB disease using days between the date of study enrollment and the date of participant abnormal chest imaging.

#### Comparison of Tests

To summarize the performance of each test individually, we computed sensitivity, specificity, PPVs, and negative predictive value (NPVs) using paired binomial proportions and 95% CIs using a modified Jeffreys method^26^ (eAppendix in Supplement 1, simultaneous confidence rectangles). For the primary study analyses, we performed pairwise comparisons of the PPV of each test using generalized estimating equations with a log-link and independence correlation structure.^27^ For all pairwise comparisons, only observations where both results were available were used for analysis and no imputation was conducted. We report PPV ratios and 95% CIs. A ratio greater than 1 represents a larger PPV of test A compared with test B in predicting TB disease progression. Ratios less than 1 favor test B (eAppendix in Supplement 1, ratios of predictive values).

#### Sensitivity Analysis

We performed PPV sensitivity analyses in separate models varying positive TSPOT result cutoffs to include spot counts of 5, 6, or 7 and 2 models varying TST universal cutoffs of 10 and 15 mm of induration. To determine whether case definitions influenced results, models were conducted restricted to case patients who had a culture-confirmed result, pulmonary TB, progression during the first 2 years, and progression after 2 years from enrollment. To examine whether results were consistent across participant subgroups, we restricted analysis to untreated participants, close contacts, and excluded close contacts. Finally, we repeated the model with case patients with prevalent TB only and performed models combining case patients with prevalent and incident TB to evaluate results with increased sample size.

#### Incremental Predictive Values

We estimated the incremental gain of a second test result, given the result of a first test in TB disease prediction. We calculated the incremental change in predictive value for progression to incident TB of an additional concurrent test B result, given that test A was positive.^27^ The model accounted for test correlation within patients (eAppendix in Supplement 1, incremental values of tests for prediction). The incremental value is given as a ratio with statistical significance when the 95% CI does not contain 1.

#### Quantitative Values of IGRAs and Time to Progression to Incident TB Disease

Among case patients with incident TB, we explored QFT-GIT and TSPOT quantitative values, computing QFT-GIT quantitative values from the antigen value minus the nil. For TSPOT counts, we selected the highest value (panel A minus the nil vs panel B minus the nil) per the manufacturer’s instructions. We report median values and IQRs by TB disease status.

#### TBI Treatment Status

Among participants with at least 1 positive test result, we examined the effects of TBI treatment on TB disease progression. Unadjusted Cox proportional hazard regression models compared participants who started treatment or completed treatment with untreated participants. The Schoenfeld residuals test was used to test proportional hazards assumptions. Hazard ratios (HRs) and 95% CIs were obtained using the survival package in R.^28^

All analyses were conducted using R, version 4.3.1 (R Project for Statistical Computing).^29^ Data analysis was performed in June 2023.

## Results

From July 2012 to May 2017, 22 131 participants were enrolled in this study and 111 were later excluded as ineligible according to the enrollment criteria. The 22 020 eligible participants had a median age of 32 (range, 0-102) years; 51.2% were men and 48.8% were women. Of these eligible participants, 0.6% were American Indian or Alaska Native, 29.8% were Asian, 20.8% were Black, 11.9% were Hispanic, 2.1% were Native Hawaiian or Other Pacific Islander, 9.5% were White, 21.0% were of other race or ethnicity, and 5.6% did not know their race or ethnicity or declined to answer. Most participants (82.0%) were born outside the US, and 9.6% were close contacts.

Tuberculosis disease was identified in 129 participants (0.6%; case patients); 87 were classified as having prevalent TB and were excluded from primary analyses. Overall, 42 case patients (0.2%) with incident TB were identified; TB disease was not identified for 21 891 participants (99.4%; Figure). Among the 42 case patients with incident TB, 35 (83.3%) had pulmonary TB, 32 (76.2%) had a culture-confirmed result, and 19 (45.2%) had a smear-positive result (eTable 1 in Supplement 1). The most frequently reported reason for study enrollment was birthplace outside the US (17 985 [82.0%]). Incident TB was notably more common among those younger than 2 years or older than 65 years, Native Hawaiian or Other Pacific Islander individuals, close contacts of case patients with TB, and participants with chronic kidney failure or diabetes (Table 1). Characteristics of case patients with prevalent TB are described elsewhere (eTable 2 in Supplement 1).

**Figure.**
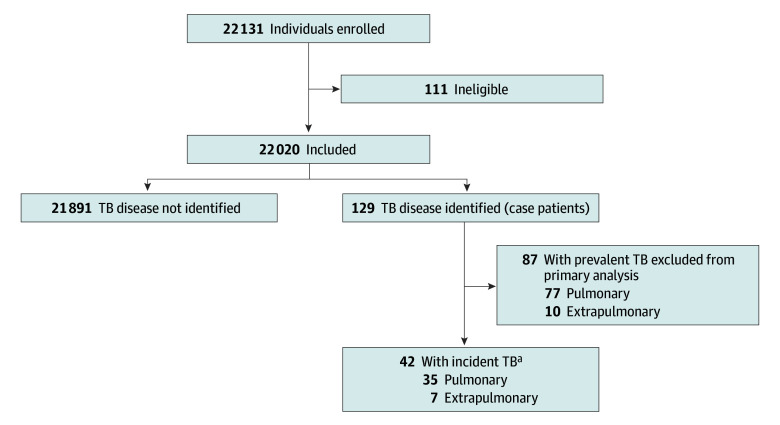
Study Population ^a^Among the 42 case patients with incident tuberculosis (TB), 35 (83.3%) had pulmonary TB, 32 (76.2%) had a culture-confirmed result, and 19 (45.2%) had a smear-positive result.

**Table 1.** Demographic and Clinical Characteristics of Participants by Incident TB Disease Status

Characteristic	No. (%) of participants		*P* value^a^
	TB not identified (21 891 [99.8])	Incident TB (42 [0.2])	
Sex^b^			
Male	11 197 (51.1)	23 (54.8)	.64
Female	10 694 (48.9)	19 (45.2)	
Age group, y			
<2	238 (1.1)	1 (2.4)	.05
2-4	756 (3.5)	0	
5-9	1379 (6.3)	0	
10-14	1537 (7.0)	5 (11.9)	
15-24	3830 (17.5)	10 (23.8)	
25-44	8478 (38.7)	12 (28.6)	
45-64	4914 (22.5)	10 (23.8)	
≥65	756 (3.5)	4 (9.5)	
Missing or unknown	3 (0.0)	0	
Race and ethnicity^c^			
American Indian or Alaska Native	126 (0.6)	0	>.99
Asian	6521 (29.8)	11 (26.2)	.61
Black or African American	4563 (20.8)	7 (16.7)	.51
Hispanic or Latino	2601 (11.9)	7 (16.7)	.34
Native Hawaiian or Other Pacific Islander	459 (2.1)	5 (11.9)	.002
White	2091 (9.6)	0	.03
Other^d^	4604 (21.0)	5 (11.9)	.15
Do not know or declined to answer	1231 (5.6)	8 (19.0)	.002
Reason for enrollment^c^			
Close contact of an individual with infectious TB	2087 (9.5)	16 (38.1)	<.001
Born outside the US^e^	17 949 (82.0)	36 (85.7)	.68
Spent >30 d in a country with high TB rates	5217 (23.8)	9 (21.4)	.71
Living with HIV	1882 (8.6)	1 (2.4)	.26
High local TBI prevalence^f^	1314 (6.0)	2 (4.8)	.43
Self-reported medical history			
Chronic kidney failure	130 (0.6)	1 (2.4)	<.001
Missing or unknown	257 (1.2)	0	
Diabetes	1113 (5.1)	11 (26.2)	<.001
Missing or unknown	266 (1.2)	0	
Immunosuppressive therapy	325 (1.5)	0	>.99
Bacille Calmette-Guérin vaccination	11 822 (60.9)	20 (54.1)	.39
Missing or unknown	2498 (11.4)	5 (11.9)	

Abbreviations: TB, tuberculosis; TBI, tuberculosis infection.

^a^ Pearson χ^2^ test or Fisher exact tests were performed.

^b^ Male and female categories include transgender participants.

^c^ Categories are not mutually exclusive and multiple categories may apply to each participant.

^d^ Not defined and often selected for multiple races or ethnicities.

^e^ Member of a local population with documented TBI prevalence of 25% or greater; includes 1045 individuals with homelessness shelter exposure.

^f^ Includes participants born outside the US enrolled under a member of a local population with documented TBI prevalence of 25% or greater.

The overall median follow-up was 6.4 (range, 0.2-8.3) years. For participants without TB disease identified, follow-up ranged from 3.5 to 8.3 years. Among the 42 case patients with incident TB, the median time to disease detection was 2.7 (range, 0.2-7.4) years, with 18 patients (42.9%) with incident TB diagnosed less than 2 years from enrollment (eFigure 1 in Supplement 1).

### Comparison of Tests

In pairwise comparisons of PPV ratios for predicting incident TB disease, both the TSPOT and QFT-GIT performed significantly better than the TST in predicting progression to TB disease (1.65 [95% CI, 1.35-2.02] and 1.47 [95% CI, 1.22-1.77], respectively). No difference in performance was detected between the 2 IGRAs (QFT-GIT vs TSPOT: 0.89 [95% CI, 0.75-1.06]; Table 2). Descriptive sensitivity, specificity, PPVs, and NPVs are reported for single tests and test result combinations (eTables 3 and 4 in Supplement 1).

**Table 2.** PPV Ratios of Tests by Pairwise Comparisons for Incident TB Disease^a^

Test^b^	PPV ratio (95% CI)			
	TST	TSPOT assay	QFT-GIT assay
TST	NA	1.65 (1.35-2.02)	1.47 (1.22-1.77)
TSPOT assay	NA	NA	0.89 (0.75-1.06)

Abbreviations: NA, not applicable; PPV, postive predictive value; QFT-GIT, QuantiFERON Gold In-Tube; TB, tuberculosis; TSPOT, T-SPOT.TB; TST, tuberculin skin test.

^a^ Values indicate the ratio of test PPVs with 95% CIs in case patients with incident TB compared with those without TB identified (horizontally across) with test B (vertically up). To note, NA is noted when comparing a test to itself or a comparison made elsewhere in the table.

^b^ Standard US cutoffs were used for the TST and QFT-GIT, and US cutoffs were used with borderlines as negative for the TSPOT.

### Sensitivity Analysis

We performed a crude sensitivity analysis by recoding TSPOT borderline values as positive, and PPV ratios remained consistent with superior TSPOT performance compared with the TST and no significant difference between the QFT-GIT and the TSPOT. The TST cutoffs were redefined as 10 and 15 mm of induration and both IGRAs continued to outperform the TST (eTable 5 in Supplement 1). When we restricted case analysis to case patients with culture-confirmed TB, pulmonary TB, early progressors, and late progressors or included both case patients with prevalent TB and with incident TB as the outcome, the conclusions remained the same. When we limited the analysis to untreated participants, close contacts, or excluded close contacts, the results remained stable, with both IGRAs outperforming the TST with PPV ratios exceeding 1 (eTable 5 in Supplement 1).

### Incremental Predictive Values

The incremental gain in PPV, given a known positive TST result, was significant for both QFT-GIT and TSPOT positive results (1.64 [95% CI, 1.40-1.93] and 1.94 [95% CI, 1.65-2.27], respectively) and negative results (0.36 [95% CI, 0.18-0.75] and 0.29 [95% CI, 0.13-0.65], respectively) (Table 3). When assessing the incremental value of a second test on the NPV of the first test, all estimates were close to 1 and not statistically significant. As a sensitivity analysis, we analyzed the incremental gain given a positive TST cutoff of 10 and 15 mm, and the results remained similar (eTable 6 in Supplement 1).

**Table 3.** Incremental Value Gained by Second Test: Change in PPV

Test A^a^	Test B	Incremental change estimate in PPV (95% CI)^b^
TST^+^	QFT^−^GIT^+^	1.64 (1.40-1.93)
TST^+^	QFT^−^GIT^−^	0.36 (0.18-0.75)
TST^+^	TSPOT^+^	1.94 (1.65-2.27)
TST^+^	TSPOT^−^	0.29 (0.13-0.65)
QFT^−^GIT^+^	TST^+^	1.10 (0.98-1.24)
QFT^−^GIT^+^	TST^−^	0.54 (0.19-1.59)
QFT^−^GIT^+^	TSPOT^+^	1.24 (1.09-1.41)
QFT^−^GIT^+^	TSPOT^−^	0.39 (0.13-1.12)
TSPOT^+^	TST^+^	1.10 (1.02-1.18)
TSPOT^+^	TST^−^	0.31 (0.05-2.13)
TSPOT^+^	QFT^−^GIT^+^	1.08 (1.00-1.17)
TSPOT^+^	QFT^−^GIT^−^	0.34 (0.05-2.33)

Abbreviations: PPV, positive predictive value; QFT-GIT, QuantiFERON Gold In-Tube; TB, tuberculosis; TSPOT, T-SPOT.TB; TST, tuberculin skin test.

^a^ Standard US cutoffs were used for the TST and QFT-GIT, and US cutoffs were used with borderlines as negative for the TSPOT. Plus signs and minus signs indicate positive and negative results, respectively.

^b^ Estimates of the test B result, given the known results of test A in predicting incident TB.

### Quantitative IGRA Values and Time to Progression to Incident TB Disease

The median quantitative QFT-GIT at enrollment was 1.38 (IQR, 0.26-7.44) IU/mL for case patients with incident TB and 0.02 (IQR, 0-0.3) IU/mL for those for whom TB was not identified. Similarly, the TSPOT median count at enrollment was 24 (IQR, 3.5-48) for case patients with incident TB and 0 (IQR, 0-4) for those without TB identified (eFigure 2 in Supplement 1).

### TBI Treatment Status

Participants with at least 1 positive test result were 8 times more likely to progress to incident TB than those without a single positive result (HR, 7.83 [95% CI, 3.30-18.6]). Among those with a positive test result, completing treatment was significantly protective in preventing incident TB disease compared with untreated participants (HR, 0.40 [0.17-0.96]; Table 4).

**Table 4.** TBI Treatment Status for Participants With at Least 1 Positive Test Result by TB Disease Status

Characteristic	No. (%) of participants		Annual incidence per 1000 person-years	HR (95% CI)
	Incident TB	No TB identified		
≥1 Positive test result	36/42 (85.7)	9417/21 795 (43.2)^a^	.0028	7.83 (3.30-18.60)
TBI treatment status^b^				
Untreated	23/35 (65.7)	5286/9268 (57.0)	.0098	1 [Reference]
Started	12/35 (34.2)	3982/9268 (42.9)	.0051	0.64 (0.33-1.25)
Completed	6/35 (17.1)	3119/9260 (33.7)	.0025	0.40 (0.17-0.96)

Abbreviations: HR, hazard ratio; TB, tuberculosis; TBI, tuberculosis infection.

^a^ Excluding 96 participants who were missing all 3 test results (all had no TB identified).

^b^ Excluding 158 participants who were missing TBI treatment data (1 case patient with incident TB and 157 with no TB identified). Separate Cox proportional hazard regression models were run for started and completed treatment compared with the referent group of untreated participants.

## Discussion

We investigated the ability of 3 FDA-approved TBI tests to predict future TB disease in a large prospective cohort enrolled in TB clinic settings in the US. The cohort was composed of predominantly young individuals born outside the US. When the TST and IGRAs were compared directly, IGRAs showed superior performance in predicting incident TB disease. Both the QFT-GIT and TSPOT assays had higher PPVs than the TST, although the PPVs of the 2 IGRAs were similar. These findings remained consistent throughout sensitivity analyses of subgroups, timing of disease diagnosis, TBI treatment, and TSPOT cut points. This study provides updated information regarding the added benefit of IGRAs over the TST, even when a positive TST result is known, and supports the benefits of TBI treatment to prevent TB disease.

Our results are consistent with the UK Prognostic Evaluation of Diagnostic IGRAs Consortium (PREDICT) TB study conducted by Abubakar et al,^17^ showing enhanced performance of IGRAs over the TST. Although the UK PREDICT investigators reported stronger performance for the TSPOT, our study did not detect any performance difference between the TSPOT and the QFT-GIT. One reasonable explanation for this difference is that international cutoffs for spot counts were used in the UK PREDICT TB study. However, when we evaluated borderline TSPOT values as positive, our results remained unchanged. Other plausible explanations include differences in sample size and TB incidence between the studies. Although our study is (to our knowledge) the largest cohort of people at high risk of TB in a low-incidence setting to date, with more than 22 000 participants enrolled, only 42 case patients with incident TB disease were identified compared with 97 case patients with incident TB disease from a cohort of 9610 in the UK PREDICT TB study. To assess the effect of TB case numbers on our results, we ran models adding case patients with prevalent TB but detected no difference between IGRAs. This study strengthens evidence supporting the use of IGRAs. Other studies comparing these tests in TB disease prediction have been limited to settings different from the US in their populations, TB incidence, and TBI treatment frequency.^17,30,31^ Most new TB cases in the US are among populations born outside the US, as reflected in this study. In the US setting and our cohort born predominantly outside the US, we found no advantage of one IGRA over the other.

Our findings support current recommendations published in 2017 by the ATS, IDSA, and CDC, in which IGRAs are preferred over the TST for TBI testing and for informing treatment decisions.^19^ Modeling studies provide evidence supporting the cost-effectiveness of using an IGRA.^19,32,33,34,35^ Despite recommendations to test with an IGRA, situations in which a TST is performed as a screening tool still occur. A confirmatory IGRA may provide more confidence in clinical decisions to promote TBI treatment to prevent progression to TB disease. Our results suggest that higher quantitative values of IGRAs may be associated with progression to TB disease.^36^ A final important study result was further evidence for TBI treatment: our study identified a statistically significant protective effect of completing TBI treatment, showing a 60% reduction (HR, 0.40 [95% CI, 0.17-0.96]) in progression to TB disease among treated individuals compared with untreated individuals. These findings suggest treatment effects under clinical programmatic conditions and further support current TB elimination strategies in the US to increase TBI treatment in people with evidence of TBI.

### Limitations

There are several limitations to this study. The QFT-Plus (Qiagen) is now the current assay in use, replacing the QFT-GIT. The QFT-Plus assay was unavailable throughout our study; however, the QFT-GIT has been shown to be comparable to the QFT-Plus.^37,38,39^ We were unable to exclude participants with a history of treated TB disease. We cannot describe the tests’ ability to predict disease over an individual’s lifetime, as follow-up time for TB disease progression was variable and results were confined to disease prediction within the study’s maximum follow-up of 8.3 years. In addition, this study was conducted among individuals at high risk of TB disease and may not be generalizable to all people in the US at risk of developing TB disease. For participants with a negative test result at enrollment, we relied on registry matches for case identification and could not assess subsequent test result changes or new exposures. Therefore, misclassification of incident and prevalent TB disease was possible. However, sensitivity analyses revealed that classification did not change our findings on test comparison. Although we are limited in our ability to assess new exposures and may have missed some cases, this study demonstrates the utility of using registry matches: we identified 5 case patients with incident TB among participants with triple-negative test results and 24 case patients with incident TB among participants with positive test results who developed TB disease after the 2-year active follow-up period through registry matches. State-level registry matches, however, could not capture disease among participants who moved to another state. Since most participants had no TB disease detected, it is unlikely that a few misclassified cases would affect the results. The clinicians who cared for patients were aware of the initial test results, and it is possible that individuals with a positive IGRA may have been more likely to undergo future testing (eg, culture) to detect TB disease, which may have favored IGRA in disease prediction. Finally, the very low incidence of TB disease limited the analytic capacity for multivariable risk factor models to assess differences in test performance in specific subgroups.

## Conclusions

In this diagnostic study assessing the predictive value of 3 tests, IGRAs demonstrated superior performance to TSTs for predicting incident TB. The uncertain ability of FDA-approved tests to predict development of TB disease is one factor that has held back adoption of recommended routine testing of individuals at risk of TB. Specifically, it has been unclear whether IGRAs outperform the TST and whether it is time to retire the TST, which is more than 100 years old. The findings of this large prospective study support the use of IGRAs over the TST for TB disease prediction, even when TST results exist, and support the benefits of a single testing strategy using an IGRA test. While this study provided evidence to guide testing strategies given currently available tests, it also underscored the need for new predictive tests to be developed for clinical use. Advancing TB elimination requires tests that can more accurately tell us who will develop TB disease and who will not.

## Notes

Supplement 1. **eAppendix.** Explanation of Statistical Methods
eReferences
**eTable 1.** Clinical and Laboratory Characteristics of Tuberculosis Cases by Case Status **eTable 2.** Demographic and Clinical Characteristics for Participants by Prevalent Tuberculosis (TB) Disease Status **eFigure 1.** Time to Progression to Incident Tuberculosis (TB) Disease (n = 42) **eTable 3.** Sensitivity, Specificity, Positive Predictive Value, and Negative Predictive Value for Individual Tests **eTable 4.** Test Combinations by Tuberculosis (TB) Disease Status **eTable 5.** Sensitivity Analysis: Pairwise Comparisons of Positive Predictive Values (PPVs) for Tuberculosis (TB) Disease **eTable 6.** Incremental Value Gained by Second Test Compared to Varying Initial TST Cutoff: Change in Positive Predictive Value **eFigure 2.** Quantitative Interferon-γ Release Assay Values and Timing to Incident Tuberculosis (TB) Disease

Supplement 2.
Nonauthor Collaborators

Supplement 3.
Data Sharing Statement
