# Error in Results and Figure

**DOI:** 10.1001/jamanetworkopen.2024.17484

**Published:** 2024-05-20

In the Original Investigation titled “Comparison of Tuberculin Skin Testing and Interferon-γ Release Assays in Predicting Tuberculosis Disease,”^1^ published April 3, 2024, the number of patients with pulmonary tuburculousis was incorrectly given as 5 instead of 35 in the Results section and in the caption of the Figure. This article has been corrected.
