# Supplementary material for: Comparison of Tuberculin Skin Testing and Interferon-γ Release Assays in Predicting Tuberculosis Disease
Source: JAMA Netw Open. 2024 Apr 3;7(4):e244769. doi: 10.1001/jamanetworkopen.2024.4769 (PMC10993073; doi:10.1001/jamanetworkopen.2024.4769)
Supplement: Supplement 1. — eAppendix. Explanation of Statistical Methods eReferences eTable 1. Clinical and Laboratory Characteristics of Tuberculosis Cases by Case Status eTable 2. Demographic and Clinical Characteristics for Participants by Prevalent Tuberculosis (TB) Disease Status eFigure 1. Time to Progression to Incident Tuberculosis (TB) Disease (n = 42) eTable 3. Sensitivity, Specificity, Positive Predictive Value, and Negative Predictive Value for Individual Tests eTable 4. Test Combinations by Tuberculosis (TB) Disease Status eTable 5. Sensitivity Analysis: Pairwise Comparisons of Positive Predictive Values (PPVs) for Tuberculosis (TB) Disease eTable 6. Incremental Value Gained by Second Test Compared to Varying Initial TST Cutoff: Change in Positive Predictive Value eFigure 2. Quantitative Interferon-γ Release Assay Values and Timing to Incident Tuberculosis (TB) Disease [file jamanetwopen-e244769-s001.pdf]

Supplementary Online Content

Ayers T, Hill AN, Raykin J, et al. Comparison of tuberculin skin testing and interferon-γ release assays in predicting tuberculosis disease. *JAMA Netw Open*. 2024;7(4):e244769. doi:10.1001/jamanetworkopen.2024.4769

eAppendix. Explanation of Statistical Methods

eReferences

eTable 1. Clinical and Laboratory Characteristics of Tuberculosis Cases by Case Status

eTable 2. Demographic and Clinical Characteristics for Participants by Prevalent Tuberculosis (TB) Disease Status

eFigure 1. Time to Progression to Incident Tuberculosis (TB) Disease (n = 42)

eTable 3. Sensitivity, Specificity, Positive Predictive Value, and Negative Predictive Value for Individual Tests

eTable 4. Test Combinations by Tuberculosis (TB) Disease Status

eTable 5. Sensitivity Analysis: Pairwise Comparisons of Positive Predictive Values (PPVs) for Tuberculosis (TB) Disease

eTable 6. Incremental Value Gained by Second Test Compared to Varying Initial TST Cutoff: Change in Positive Predictive Value

eFigure 2. Quantitative Interferon-γ Release Assay Values and Timing to Incident Tuberculosis (TB) Disease

This supplementary material has been provided by the authors to give readers additional information about their work.

© 2024 Ayers T et al. *JAMA Network Open.*

eAppendix. Explanation of Statistical Methods

eMethods

Simultaneous confidence rectangles – for sensitivity, specificity, PPV, and NPV

Paired binomial proportions and simultaneous 95% confidence rectangles were estimated by modified Jeffreys intervals using R DescTools package.[1] These intervals were proposed and recommended by Brown *et al.* (2001)[2] to improve coverage of Jeffreys prior intervals for estimates near the boundary (PPVs near 0 and NPVs near 1 in this study). Simultaneous confidence rectangles were obtained by computing $\sqrt{95\%}$ univariate confidence intervals for each test-specific pair of sensitivity, specificity, positive predictive value (PPV), and negative predictive value (NPV). As data from TB and non-TB subjects are independent, and within-test positive and negative subjects in a cohort study are independent, the overall confidence level for these rectangles is $\sqrt{95\%} \times \sqrt{95\%} = 95\%$.

Ratios of predictive values

Pairwise ratios of PPVs were estimated via a log-binomial GEE model using R geeM package.[3] Data were arranged in long format over participant ID. The regression equation takes the form

$$\log \Pr[D = Y \mid Y, Test] = \alpha_0 + \alpha_1 Y + \alpha_2 X_{Test} + \alpha_3 Y X_{Test}$$

where D indicates progression status (progressed or did not, the latter as referent), X_{Test} indicates test type (TST, QFT-GIT, T-SPOT), and Y denotes test result (positive or negative, the latter as referent). An independence working covariance matrix was used to ensure consistent parameter estimates[4,5] and participant ID was the GEE clustering variable.

Agreement of progression status and each test result was the outcome, and test type and test result were predictors with an interaction term between them. The model enables simultaneous estimation of ratios of PPVs and NPVs.

Specifically, taking TST as the referent for test result, we index the regression parameters for X_{Test} as α_{2Q} for QFT-GIT and α_{2T} for T-SPOT; similarly α_{3Q} and α_{3T}. It follows that

$$PPV(TST) = \exp(\alpha_0 + \alpha_1),$$

$$PPV(QFT) = \exp(\alpha_0 + \alpha_1 + \alpha_{2Q} + \alpha_{3Q}),$$

$$PPV(TSPOT) = \exp(\alpha_0 + \alpha_1 + \alpha_{2T} + \alpha_{3T}).$$

This implies that the ratios of PPVs, where *rPPV(A,B)* denotes the ratio *PPV(A)*/*PPV(B)* of test *B* to test *A*, are given by

$$rPPV(QFT, TST) = \exp(\alpha_{2Q} + \alpha_{3Q}),$$

$$rPPV(TSPOT, TST) = \exp(\alpha_{2T} + \alpha_{3T}).$$

Standard errors and confidence intervals may be derived by linear transformations of the robust sandwich regression variance-covariance matrix in the usual fashion. The ratio $rPPV(TSPOT, QFT)$ is estimated as

$$\exp(\alpha_{2T} - \alpha_{2Q} + \alpha_{3T} - \alpha_{3Q}).$$

NPVs can be estimated similarly. For example,

$$rNPV(QFT, TST) = \exp(\alpha_{2Q}).$$

Our model differs from the one employed by Abubakar *et al.* (2018)[6] who estimate ratios of positive diagnostic likelihood ratios. These are mathematically equivalent to ratios of odds ratios of PPVs. As progression in our study is a rare outcome, the latter closely approximates the ratio of PPVs. When we ran the corresponding models to estimate odds ratios, results agreed with those presented in the main manuscript.

© 2024 Ayers T et al. *JAMA Network Open.*

Incremental values of tests for prediction

In estimating incremental value of a second test over a first, we adopted another GEE model by Pepe (2004, p.59).[7] The model is restricted to those subjects who tested positive on the first test and has as its outcome progression to TB disease, conditioned on the outcome of both tests or on the first only, made possible by arrangement again of the data in long format over subject ID. Independent variables are a binary label variable indicating whether or not to include the result of the second test, and the result itself of the second test. Label is the main effect, and the model includes an interaction term of label and second test result, but specifically excludes the second test result as a main effect. This enables comparison of the PPV of the first with the predictive value when also incorporating the second test result. The incremental value is given as the ratio of the latter to the former and statistical significance is attained when the confidence interval does not contain 1. The incremental value is the ratio of the predictive value of progression to incident TB disease given the results of test A (positive) and test B (positive or negative) to the PPV of test A on its own. We report separate incremental values, and 95% CIs, when test B is positive and when test B is negative. An incremental value when test B is positive whose lower confidence limit is > 1 indicates a greater risk of incident TB disease from a double positive on both tests. Similarly, an upper confidence limit for the incremental value which is < 1 when test B is negative indicates a decreased risk of incident TB disease compared to a positive result on test A alone.

To compare the additional information gained by test *B* after a positive result on test *A*, the data are restricted to those participants who are positive on test *A*. Two rows of data are assigned for each participant with a binary label variable X_L indicating if the result for test *B* should be included for that participant ($X_L = 1$ if included, 0 if not). The model takes the form

$$\log \Pr[D = 1 \mid Y_A = 1, Y_B, X_L] = \beta_0 + \beta_1 X_L + \beta_2 X_L Y_B,$$

where *D* indicates progression status (progressed = 1, did not = 0), and Y_A and Y_B denote the results of test *A* (positive = 1), and test *B* respectively. The GEE model is run with independence working covariance matrix and clustering on participant ID. With this formulation, e^{β_0} is the PPV of test *A* on its own ($X_L = 0$). When $X_L = 1$, the result of test *B* is included and

$$\exp(\beta_0 + \beta_1) = \Pr[D = 1 \mid Y_A = 1, Y_B = 0],$$

$$\exp(\beta_0 + \beta_1 + \beta_2) = \Pr[D = 1 \mid Y_A = 1, Y_B = 1].$$

The first expression gives the predictive value of test *A* positive and test *B* negative; the second the predictive value when both tests are positive. Incremental values of these quantities relative to $PPV(A) = \Pr[D = 1 \mid Y_A = 1]$ are

$$\exp(\beta_1) = \frac{\Pr[D = 1 \mid Y_A = 1, Y_B = 0]}{\Pr[D = 1 \mid Y_A = 1]},$$

$$\exp(\beta_1 + \beta_2) = \frac{\Pr[D = 1 \mid Y_A = 1, Y_B = 1]}{\Pr[D = 1 \mid Y_A = 1]}$$

These quantify the relative change in predictive value for TB disease of a positive test *A* with an additional negative or positive result for test *B*, respectively. The regression framework allows estimation of robust sandwich standard errors and confidence intervals for these quantities.

Incremental values for NPV of test *A* arising from test *B* results can be modeled similarly, with the data now restricted to negative results for *A* and the outcome on the left hand side of the regression equation as $\log \Pr[D = 0 \mid Y_A = 0, Y_B, X_L]$.

© 2024 Ayers T et al. *JAMA Network Open.*

eReferences

1. Signorell A, et al. DescTools: Tools for descriptive statistics. R package version 0.99.46. 2022. https://cran.r-project.org/package=DescTools
2. Brown LD, Cai TT, DasGupta A. Interval Estimation for a Binomial Proportion. *Statistical Science*. 2001;16(2):101-117
3. McDaniel LS, Henderson NC, Rathouz PJ. Fast Pure R Implementation of GEE: Application of the `Matrix Package. *R j*. Jun 2013;5(1):181-187. https://journal.r-project.org/archive/2013-1/mcdaniel-henderson-rathouz.pdf
4. Pepe MS, Anderson GL. A cautionary note on inference for marginal regression models with longitudinal data and general correlated response data. *Communications in Statistics - Simulation and Computation*. 1994/01/01 1994;23(4):939-951. doi:10.1080/03610919408813210
5. Pepe MS, Whitaker RC, Seidel K. Estimating and comparing univariate associations with application to the prediction of adult obesity. *Stat Med*. Jan 30 1999;18(2):163-73. doi:10.1002/(sici)1097-0258(19990130)18:2<163::aid-sim11>3.0.co;2-f
6. Abubakar I, Drobniewski F, Southern J, et al. Prognostic value of interferon-γ release assays and tuberculin skin test in predicting the development of active tuberculosis (UK PREDICT TB): a prospective cohort study. *The Lancet Infectious diseases*. Oct 2018;18(10):1077-1087. doi:10.1016/s1473-3099(18)30355-4
7. Pepe MS. *The statistical evaluation of medical tests for classification and prediction*. paperback ed. Oxford University Press; 2004

© 2024 Ayers T et al. *JAMA Network Open.*

eTable 1. Clinical and Laboratory Characteristics of Tuberculosis Cases by Case Status

Characteristics	Incident cases (n= 42)		Prevalent cases (n=87)	
**Sites of Disease**				
**Pulmonary**	35	83%	77	89%
**Extrapulmonary**	7	17%	10	11%
Pleural	4	10%	3	3%
Lymphatic	2	5%	6	7%
Bone and/or joint	1	2%	3	3%
Peritoneal	1	2%	0	0%
Other: Spinal abscess	1	2%	0	0%
**Extent of Disease**				
Both pulmonary and extra pulmonary	4	10%	3	3%
Culture-positive	32	76%	52	60%
Sputum smear-positive	19	45%	16	18%

© 2024 Ayers T et al. *JAMA Network Open.*

eTable 2. Demographic and Clinical Characteristics for Participants by Prevalent Tuberculosis (TB) Disease Status

Characteristics	TB not identified		Prevalent TB		P-value[a]
	N	(%)	N	(%)	
**All participants**	21,891	99.8	87	0.4	
**Gender**[b]					0.04
Male	11,197	51.1	54	62.1	
Female	10,694	48.9	33	37.9	
**Age group (years)**					0.17
< 2	238	1.1	2	2.3	
2–4	756	3.5	4	3.4	
5–9	1,379	6.3	5	6.9	
10–14	1,537	7.0	2	2.3	
15–24	3,830	17.5	12	12.6	
25–44	8,478	38.7	39	43.7	
45–64	4,914	22.5	17	21.8	
≥ 65	756	3.5	6	6.9	
Missing	3	0.0	0	0.0	
**Race or ethnicity**[c]					
American Indian/Alaska Native	126	0.6	2	2.3	0.09
Asian	6,521	29.8	28	32.2	0.63
Black/African American	4,563	20.8	16	18.4	0.57
White/Caucasian	2,091	9.6	2	2.3	0.02
Native Hawaiian/Pacific Islander	459	2.1	2	2.3	0.71
Hispanic Latino	2,601	11.9	9	10.3	0.66
Other	4,604	21	24	27.6	0.13
Don’t know/declined	1,231	5.6	5	5.7	0.82
**Reason for enrollment**[c]					
Close contact to an infectious TB case	2,087	9.5	18	20.7	<.001
Non-U.S.-born[d]	17,949	82.0	70	80.5	0.46
Spent > 30 days in a country with high TB rates	5,217	23.8	21	24.1	
HIV-positive	1,882	8.6	3	3.4	0.09
High local LTBI prevalence[e]	1,314	6.0	0	0	
**Self-reported medical history**					
Chronic kidney failure	130	0.6	2	2.3	<.001
Missing/unknown	257	1.2	0	0	
Diabetes	1,113	5.1	12	13.8	<.001
Missing/unknown	266	1.2	0	0	
Immunosuppressive therapy	325	1.5	1	1.1	>0.99
Bacille Calmette-Guérin vaccinated	11,822	60.9	52	59.8	0.46
Missing/unknown	2,498	11.4	7	8	

[a] Pearson's Chi-square test or Fisher's exact tests were performed. Gender and Age were tested with an overall test since categories were mutually exclusive. For all other characteristics, which were not mutually exclusive, individual categories were treated as separate binary categories as the presence or absence of a characteristic.
[b] Male and Female categories include transgender.
[c] Categories are not mutually exclusive and multiple categories may apply to each participant.
[d] Includes non-US-born participants enrolled under member of a local population with documented TB infection prevalence ≥ 25%
[e] Member of a local population with documented LTBI prevalence ≥25%; includes 1,045 persons with homeless shelter exposure.

© 2024 Ayers T et al. *JAMA Network Open.*

eFigure 1. Time to Progression to Incident Tuberculosis (TB) Disease (n = 42)

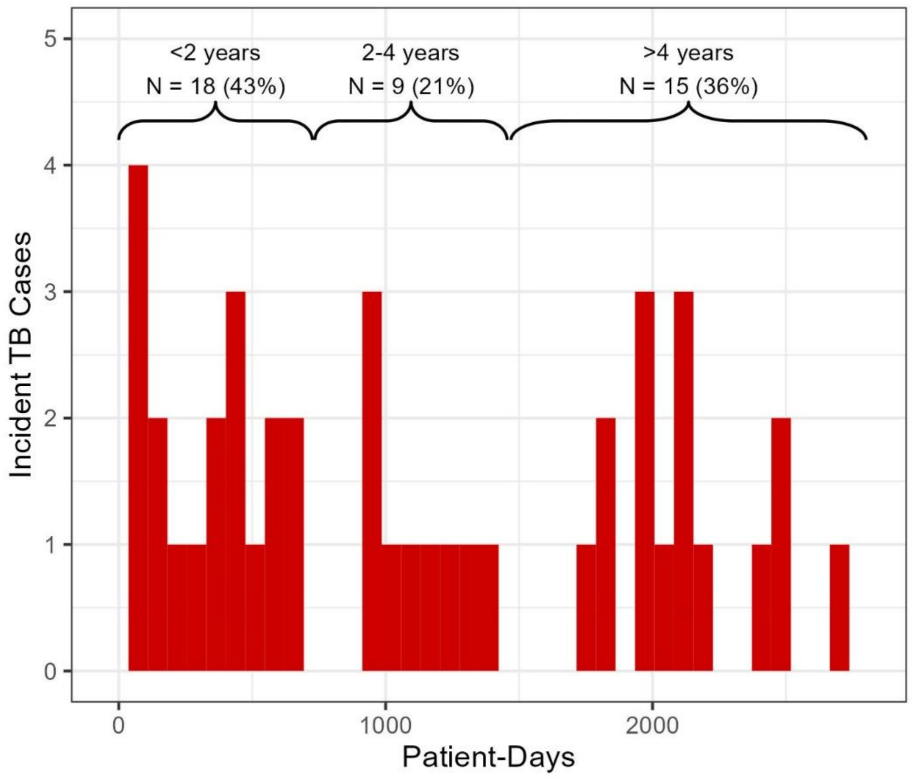

eTable 3. Sensitivity, Specificity, Positive Predictive Value, and Negative Predictive Value for Individual Tests[a,b]

	**TST** (95% CI)	**QFT-GIT** (95% CI)	**TSPOT** (95% CI)
**Sensitivity**	0.7857 (0.6240, 0.89995)	0.7143 (0.5453, 0.8476)	0.6842 (0.5046, 0.8301)
**Specificity**	0.6140 (0.6066, 0.6215)	0.7634 (0.7569, 0.7698)	0.8094 (0.8033, 0.8155)
**Positive predictive value**	0.0040 (0.0027, 0.0058)	0.0059 (0.0038, 0.0086)	0.0066 (0.0041, 0.01007)
**Negative predictive value**	0.9993 (0.9987, 0.9997)	0.9993 (0.9987, 0.9996)	0.9993 (0.9987, 0.9996)

[a] The following test abbreviations are used: TST (Tuberculin Skin Test, using standard US cutoffs), QFT-GIT (QuantiFERON- Gold-in-Tube, using standard US cutoffs), and TSPOT (T-SPOT.TB, using US cutoffs with borderlines as negative).
[b] Simultaneous 95% confidence rectangles are given for each pair of sensitivity and specificity and for positive predictive value and negative predictive value corresponding to each test.

eTable 4. Test Combinations by Tuberculosis (TB) Disease Status[a,b]

Test combinations	TB not identified [c]		Incident [c]		Prevalent [c]	
	n	(%)[d]	n	(%)[d]	n	(%)[d]
No test results available	96		0		0	
Single test results						
TST+	8,231	(38.6)	33	(78.6)	72	(84.7)
Total TST results	21,326		42		85	
Missing TST	565		0		2	
QFT-GIT +	5,078	(23.7)	30	(71.4)	70	(81.4)
Total QFT-GIT results	21,461		42		86	
Missing QFT-GIT	430		0		1	
TSPOT+	3,917	(19.1)	26	(68.4)	55	(87.1)
Total TSPOT results	20,555		38		82	
Missing TSPOT	1336		4		5	
3 test combinations - total	*19,900*		*38*		*80*	
Triple positive (+++)	3,042	(15.3)	24	(63.2)	53	(66.3)
Triple negative (−−−)	11,181	(56.2)	5	(13.2)	7	(8.8)
Isolated TST+ (+−−)	3,618	(18.2)	4	(10.5)	6	(7.5)
Isolated QFT-GIT + (−+−)	484	(2.4)	2	(5.3)	4	(5.0)
Isolated TSPOT+ (−−+)	143	(0.7)	0	(0.0)	0	(0.0)
TST+ & QFT-GIT + (++−)	817	(4.1)	1	(2.6)	8	(10.0)
Both IGRAs+ (−++)	328	(1.6)	1	(2.6)	1	(1.3)
TST+ & TSPOT+ (+−+)	287	(1.4)	1	(2.6)	1	(1.3)

[a] Incident cases were diagnosed greater than 30 days from enrollment or had documented change in diagnostic images (chest x-ray or CT-Scan) from enrollment. Prevalent cases were diagnosed less than or equal to 30 days of enrollment or did not have a change in diagnostic images.
[b] The following test abbreviations are used: TST (Tuberculin Skin Test, using standard US cutoffs), QFT-GIT (QuantiFERON- Gold-in-Tube, using standard US cutoffs), and TSPOT (T-SPOT.TB, using US cutoffs with borderlines as negative).
[c] There were a total of 21,891 participants with no TB identified, 42 participants with incident TB, and 87 participants with prevalent TB.
[d] Percentages are calculated using the total results (rows with no percentage) as the denominator.

© 2024 Ayers T et al. *JAMA Network Open.*

eTable 5. Sensitivity Analysis: Pairwise Comparisons of Positive Predictive Values (PPVs) for Tuberculosis (TB) Disease[a,b,c]

	TSPOT vs. TST (95% CI)	QFT-GIT vs. TST (95% CI)	QFT-GIT vs. TSPOT (95% CI)
**Incident TB** (outcome = 42)	1.65 (1.35–2.02)[d]	1.47 (1.22–1.77)[d]	0.89 (0.75–1.06)
**Alternative test cut-offs**			
TSPOT Borderline[e] Positive	1.44 (1.17–1.77)[d]	1.47 (1.22–1.77)[d]	1.02 (0.88–1.20)
TST10[f]	1.61 (1.32–1.97)[d]	1.44 (1.19–1.73)[d]	0.89 (0.75–1.06)
TST15[g]	1.31 (0.98–1.75)	1.17 (0.89–1.52)	0.89 (0.75–1.06)
**Sub-analysis**			
Culture confirmed (outcome = 32)	1.71 (1.38–2.11)[d]	1.56 (1.28–1.90)[d]	0.91 (0.75–1.11)
Pulmonary (outcome = 35)	1.66 (1.34–2.06)[d]	1.45 (1.17–1.80)[d]	0.87 (0.73–1.04)
Progressed < 2 years (outcome = 18)	1.61 (1.20–2.18)[d]	1.74 (1.36–2.24)[d]	1.08 (0.78–1.50)
Progressed after 2 years (outcome = 24)	1.68 (1.28–2.20)[d]	1.30 (0.99–1.69)	0.77 (0.65–0.92)
Untreated[h] (n= 5,326; outcome = 23)	2.09 (1.54–2.83)[d]	1.85 (1.38–2.49)[d]	0.89 (0.67–1.18)
Close contacts (n= 2,121; outcome =16)	1.91 (1.54–2.37)[d]	1.95 (1.50–2.52)[d]	1.02 (0.74–1.41)
Other high-risk (n= 19,899; outcome = 26)[i]	1.55 (1.17–2.05)[d]	1.27 (0.98–1.63)	0.82 (0.67–0.99)
**Prevalent TB** (outcome = 87)	1.60 (1.39–1.83)[d]	1.57 (1.41–1.74)[d]	0.98 (0.86–1.12)
**All TB Disease** (outcome = 129)	1.61 (1.44–1.80)[d]	1.53 (1.40–1.68)[d]	0.95 (0.86–1.06)

[a] The following test abbreviations are used: TST (Tuberculin Skin Test, using standard US cutoffs), QFT-GIT (QuantiFERON- Gold-in-Tube, using standard US cutoffs), and TSPOT (T-SPOT.TB, using US cutoffs with borderlines as negative).
[b] Positive Predictive value (PPV). Values indicate the ratio of test positivity rates (with 95% CI and p values) in participants who progressed to active tuberculosis compared with those who did not comparing test A with test B. A value above 1 indicates a positive result.
on test A is a stronger predictor of progression to tuberculosis than a positive result on test B.
[c] Unless indicated otherwise, standard US cut-offs are applied for all tests
[d] Indicates statistical significance.
[e] Borderline results for TPOT as defined as having spot counts of 5,6, and 7 (n=777) categorized as positive result.
[f] TST results redefined as induration of ≥10mm as positive and <10mm as negative
[g] TST results redefined as induration of ≥15mm as positive and <15mm as negative
[h] Among participants with at least 1 test positive, treatment data available and did not start treatment; (Incident TB= 23, TB not Identified= 5286).
[i] Excludes all participants identified as close contacts (≥ 8 hours in a week) to an individual with smear-positive TB disease

© 2024 Ayers T et al. *JAMA Network Open.*

eTable 6. Incremental Value Gained by Second Test Compared to Varying Initial TST Cutoff: Change in Positive Predictive Value[a,b]

Test A	Test B	Incremental Change Estimate (95% CI) (statistically significant in bold)
**TST10+**	**QFT-GIT +**	**1.63 (1.39–1.91)**
**TST10+**	**QFT-GIT −**	**0.37 (0.18–0.75)**
**TST10+**	**TSPOT+**	**1.91 (1.63–2.25)**
**TST10+**	**TSPOT −**	**0.30 (0.13–0.66)**
**TST15+**	**QFT-GIT +**	**1.38 (1.17–1.61)**
TST15+	QFT-GIT −	0.35 (0.12–1.02)
**TST15+**	**TSPOT+**	**1.53 (1.28–1.82)**
TST15+	TSPOT −	0.32 (0.11–0.92)

[a] The following test abbreviations are used: TST (Tuberculin Skin Test; TST10 uses indurations of ≥10mm as positive, TST15 uses indurations of ≥15mm as positive), QFT-GIT (QuantiFERON- Gold-in-Tube, using standard US cutoffs), and TSPOT (TT-SPOT.TB,using US cutoffs with borderlines as negative).
[b] Positive Predictive Value (PPV). Estimates of 'Test B' result, given the known results of 'Test A' in predicting Incident TB.

© 2024 Ayers T et al. *JAMA Network Open.*

eFigure 2. Quantitative Interferon-γ Release Assay Values and Timing to Incident Tuberculosis (TB) Disease[a,b,c]

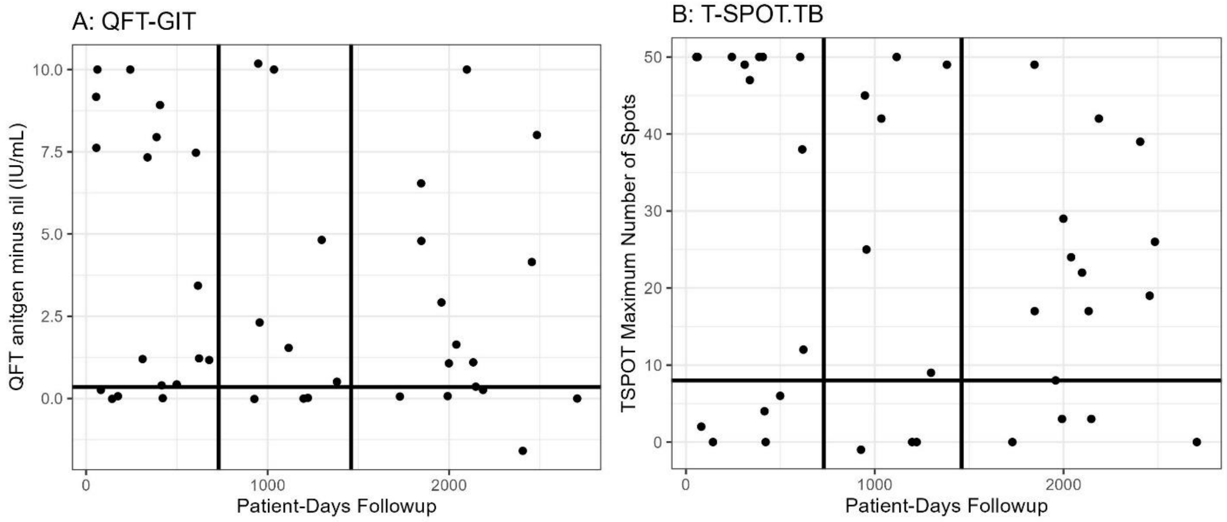

**QFT-GIT**	**Median IU/mL**	**IQR**
Incident overall (N=42)	1.38	(0.26 -7.44)
Time to progression		
less than 2 years (n= 18)	2.33	(0.40 -7.86)
2-4 years (n= 9)	1.54	(0.02 -4.82)
Late >4 years (n=15)	1.1	(0.17 -4.47)
Prevalent TB (n- 87)	1.94	(0.45- 5.60)
No TB (m= 21898)	0.02	(0 -0.3)

**TSPOT**	**Median spot count**	**IQR**
Incident overall (N=42)	24	(3.5 -48)
Time to progression		
less than 2 years (n= 18)	47	(5-50)
2-4 years (n=9)	25	(0-45)
Late >4 years (n=15)	19	(5.5-27.5)
Prevalent TB(n=87)	23	(6-50)
No TB (n= 21898)	0	(0-4)

[a] The following test abbreviations are used: QFT-GIT (QuantiFERON- Gold-in-Tube, using standard US cutoffs), and TSPOT (T-SPOT.TB, using US cutoffs with borderlines as negative).
[b] Quantitative values of QFT-GIT are based on the QFT Antigen minus the Nil. Quantitative TSPOT counts were calculated as Panel A or Panel B (whichever is the largest) minus the nil value.
[c] The vertical lines in graphs A and B are at 2 and 4 years.

© 2024 Ayers T et al. *JAMA Network Open.*
