# Supplementary material for: Comparison of Tuberculin Skin Testing and Interferon-γ Release Assays in Predicting Tuberculosis Disease
Source: JAMA Netw Open. 2024 Apr 3;7(4):e244769. doi: 10.1001/jamanetworkopen.2024.4769 (PMC10993073; doi:10.1001/jamanetworkopen.2024.4769)
Supplement: Supplement 2. — Nonauthor Collaborators [file jamanetwopen-e244769-s002.pdf]

Supplement 2. Nonauthor Collaborators
*First name, last name, and suffix (if applicable) are required and will appear in PubMed.

***Group Name(s): CDC Tuberculosis Epidemiologic Studies Consortium**

***First Name and Middle Initial(s)**	***Last Name**	***Suffix (eg, Jr, III)**	Academic Degrees	Institution	Location (city, state/province, country)	Role or Contribution, eg, chair, principal investigator	Group (if more than 1 Group listed in the byline) and/or Subgroup (eg, Steering Committee)
Lisa	Pascopella			California Department of Public Heal	Richmond, CA	Co-PI	
Amina	Ahmed		MD	Carolinas Medical Center	Charlotte, NC	Co-PI	
Kaylynn	Aiona		MPH	Denver Health and Hospital Authorit	Denver, CO	Data Manager	
Juanita	Lovato			Denver Health and Hospital Authorit	Denver, CO	Project Coordinator	
Randall	Reves		MD	Denver Health and Hospital Authorit	Denver, CO	Co-PI	
Leti	Trujillo			Denver Health and Hospital Authorit	Denver, CO	Project Coordinator	
Richard	Garfein		MD	Department of Medicine, University of California San Diego School of Medicine	San Diego, CA	Co-PI	
Laura	Farrow			Duke University	Durham, NC	Data Manager	
Jason	Stout		MD	Duke University	Durham, NC	Co-PI	
Sofia	Zavala		MD	Duke University	Durham, NC	Investigator	
Henry	Blumberg		MD	Emory Univeristy	Atlanta GA	Co-PI	
Jane	Tapia			Emory University	Atlanta GA	Project Coordinator	
Alawode	Oladele		MD	Emory University / DeKalb County B	Atlanta GA	Co-PI	
Angela	Largen		MPH	Honolulu State Health Department	Honolulu, HI	Project Coordinator	
Susan	Dorman		MD	Johns Hopkins University	Baltimore, MD	Co-PI	
Gina	Maltas			Johns Hopkins University	Baltimore, MD	Project Coordinator	
Elizabeth	Munk			Johns Hopkins University	Baltimore, MD	Project Coordinator	
Maunank	Shah		MD	Johns Hopkins University	Baltimore, MD	Co-PI	
Aurimar	Ayala		MPH	Maricopa County Department of Pub	Phoenix, AZ	Project Coordinator	
Wendy	Cronin		PhD	Maryland Department of Health (Bal	Baltimore, MD	Co-PI	
Paul	Saleeb		MD	Maryland Department of Health (Bal	Baltimore, MD	Co-PI	
Michelle	Haas		MD	National Jewish Health	Denver, CO	Co-PI	
Yoseph	Sorri			Public Health - Seattle & King County	Seattle, WA	Project Coordinator	
Christine	Kozik			San Diego Department of Health	San Diego, CA	Project Coordinator	
Marisa	Moore		MD	San Diego Department of Health	San Diego, CA	Co-PI	
Chris	Keh		MD	San Francisco Department of Public H	San Francisco, CA	Co-PI	
Laura	Romo			San Francisco Department of Public H	San Francisco, CA	Project Coordinator	

Supplement 2. Nonauthor Collaborators
*First name, last name, and suffix (if applicable) are required and will appear in PubMed.

***First Name and Middle Initial(s)**	***Last Name**	***Suffix (eg, Jr, III)**	Academic Degrees	Institution	Location (city, state/province, country)	Role or Contribution, eg, chair, principal investigator	Group (if more than 1 Group listed in the byline) and/or Subgroup (eg, Steering Committee)
Joanne C	Li			University of Florida	Gainesville, FL	Project Coordinator	
Stephanie	Reynolds-Bigby			University of Florida	Miami, FL	Project Coordinator	
Marie Nancy	Seraphin			University of Florida	Gainesville, FL	Project Coordinator	
Amy	Board			University of North Texas Health Scie	Fort Worth, TX	Project Coordinator	
Jeremy	Gallups			University of North Texas Health Scie	Fort Worth, TX	Project Coordinator	
Erica	Stockbridge		PhD	University of North Texas Health Scie	Fort Worth, TX	Co-PI	
David	Horne		MD	University of Washington	Seattle, WA	Co-PI	
Kristen	Atchley		RN	Vanderbilt University	Nashville, TN	Project Coordinator	
Fernanda	Maruri		MPH	Vanderbilt University	Nashville, TN	Project Coordinator	
Timothy	Sterling		MD	Vanderbilt University	Nashville, TN	Co-PI	
Amy	Kerrigan		RN	Vanderbilt University Medical Center	Nashville, TN	Project Coordinator	
Alicia	Wright		MS	Vanderbilt University Medical Center	Nashville, TN	Project Coordinator	
Kursten	Lyon			Wake County Human Services	Raleigh, NC	Project Coordinator	
Debra	Turner			Wake County Human Services	Raleigh, NC	Project Coordinator	
