# Supplementary material for: Comparison of Tuberculin Skin Testing and Interferon-γ Release Assays in Predicting Tuberculosis Disease
Source: JAMA Netw Open. 2024 Apr 3;7(4):e244769. doi: 10.1001/jamanetworkopen.2024.4769 (PMC10993073; doi:10.1001/jamanetworkopen.2024.4769)
Supplement: Supplement 3. — Data Sharing Statement [file jamanetwopen-e244769-s003.pdf]

Data Sharing Statement

Ayers. Comparison of Tuberculin Skin Testing and Interferon-γ Release Assays in Predicting Tuberculosis Disease. *JAMA Netw Open*. Published April 03, 2024.
doi:10.1001/jamanetworkopen.2024.4769

Data
Data available: No

Additional Information
Explanation for why data not available: Data has not yet been released and is not available, but will be provided to the public by CDC in 2024.
